# Performance of eucalypt particleboard with the addition of farm waste

**DOI:** 10.1016/j.heliyon.2023.e22760

**Published:** 2023-11-23

**Authors:** Vinícius Borges Taquetti, Vitor Viana Silva, Izabella Luzia Silva Chaves, Rafael Gonçalves Espósito Oliveira, Fernanda Dalfiôr Maffioletti, Glaucileide Ferreira, José Paulo Costa Mendonça, Emilly Soares Gomes Silva, Fabricio Gomes Gonçalves

**Affiliations:** a Postgraduate Program in Forest Sciences, Avenue Governador Lindemberg, 216, Center, 29550-000, Jerônimo Monteiro, ES, Brazil; b Engenheiro Industrial, High Jump Process Consulting Ltda., Avenue Presidente Castelo Branco, 2525, A.402, Parque Industrial Lagoinha, 14095-000, Ribeirão Preto, SP, Brazil; c Federal University of Espírito Santo, Department of Forest and Wood Science, Avenue Governador Lindemberg, 216, Center, 29550-000, Jerônimo Monteiro, ES, Brazil

**Keywords:** Eggshell, Eucalypt, Urea-formaldehyde, Particleboard

## Abstract

Wood residues, as well as those from poultry farming, are generally disposed of irregularly, which causes damage to the environment, and thus poses a great challenge in terms of their use, a possible alternative is the manufacture of particleboards. For this, the following mechanical properties of the panels were evaluated: static bending (elasticity – MOE and rupture – MOR), internal bond strength (IB), Janka hardness (DJ), screw withdrawal resistance (SW), thickness swelling (TS), water absorption (WA), moisture, apparent density (DA) and density profile, in addition to morphological characterization by scanning electron microscope image. With the results obtained, it was possible to observe that the amount of residue influenced the density of the panel. The panel compaction ratio showed little variation (1.22–1.32), remaining close to the value considered ideal of 1.33. The percentage of waste added to the panels also influenced the increase in WA and TS, limiting the use of the panels to environments without humidity. The addition of the eggshell in the process negatively affected the mechanical characteristics of formulated particleboards, except for Janka hardness. The application of the resin together with the wood particles and eggshells can improve the mechanical properties. Based on the findings from this study, the medium-density particleboard produced can be used for making furniture and room partitioning without structural character.

## 1 Introduction

In Brazil, there is a large amount of planted forests, with eucalyptus being the main species. It presents itself as the most cultivated tree and of the greatest economic importance, both in the country and in the world [1]. The country has about 9.93 million hectares of planted forests, of which 7.53 million hectares are cultivated with eucalyptus, representing about 75.8% of the Brazilian territory [2]. The genus is widely used by the forestry and timber sector as an immediate substitute for wood from native forests and also as a raw material for various purposes, in particular, for the wood-based panel segment [3,4].

Globally, the country ranks eighth in world production of wood panels, second in cellulose production and first in charcoal production [2]. All these sectors of the wood industry are major producers of lignocellulosic waste with the possibility of developing new products and reusing waste, such as the production of particleboard panels.

This type of product emerged to supply the lack of raw material that occurred during and after the Second World War and gained space in the market mainly due to its low cost. Among the existing types of panels, the particleboard is a composite formed by particles of wood species [5,6] or other lignocellulosic material [[7], [8], [9]], incorporated with a synthetic or natural adhesive, and are consolidated through the application of heat and pressure in a specific press [10].

The wood panel production sector is of great importance to the Brazilian economy. The year 2021 ended with a production of 8.2 million cubic meters of wood panels, both for domestic consumption and export [2].

The growth of the panel sector in Brazil and in the world is highly influenced by the search for more sustainable products, with better use of different raw materials. The reuse of different lignocellulosic residues for the production of particleboard is an alternative for better disposal of these materials, in addition to being an efficient way to add value to waste [11].

The union of different organic [[12], [13], [14], [15]] and inorganic materials [16,17], if pressed under high temperature and pressure, can generate a panel resistant to physical or mechanical stress and xylophagous organisms, in addition to promoting better disposal of commercially undervalued waste through ecologically correct environmental management. Among several organic materials used in the composition of particleboards, along with wood, rice husk [15,18], sugarcane bagasse [19], wheat bran [20], coffee fiber [21], bamboo waste [22], sunflower stalks [23] and, in particular, eggshell, stand out [24].

The hen’s egg is widely consumed by the majority of the Brazilian population, with the state of Espírito Santo being the largest egg producer in Brazil, where 16.4 million hens and 3.7 million quails are raised, producing over 360 million dozens of chicken eggs and 77 million dozens of quail eggs per year [25].

The commercialization of this product generates significant economic advantages. However, Brazil produces approximately 172,000 tons of eggshell waste per year from restaurants, food industries, and even households. The average density of this material is about 1.085 g/cm³, with high calcium content, and therefore, it has the potential to be used as a raw material by the industry [[26], [27], [28], [29], [30], [31], [32], [33]]. It is important to highlight that, despite the benefits of working with this material, its improper disposal can lead to increased costs for companies [34], generating environmental problems and, consequently, damage to public health [27].

Studies on the use of eggshells in various applications have intensified, whether in replacing cement [35], remediation of boron in water [34], metal removal [36] and dyes [37], or in the production of biodiesel [38]. Eggshells have also been used to compose part of agglomerated panels together with other agricultural waste [24]. However, the performance of eggshells on the properties of wood panels has not been tested.

In this context, the objective of the study is to investigate the performance of physical and mechanical properties in particleboard panels produced with eucalyptus wood particles and the addition of eggshells in different percentages. The findings can provide information that will assist in decision-making regarding the feasibility of using these products, as well as adding value to the eggshell and providing viable alternatives for the disposal of farm waste.

## 2 Materials and methods

### 2.1 Obtaining and preparing the raw material

The research was carried out at the Laboratory of Panels of the Department of Forest and Wood Sciences at the Federal University of Espírito Santo (UFES), located in the town of Jerônimo Monteiro – ES, Brazil.

For the production of the particleboard panels, wood particles from the *Eucalyptus* genus were used, sourced from plantations in the experimental area of the Agricultural Sciences and Engineering Center at the Federal University of Espírito Santo, located in the town of Alegre – ES, Brazil. The far waste, namely eggshells, was collected after culinary and industrial use, and obtained from restaurants and domestic households.

The wood logs, after being air-dried, were unfolded into horizontal bandsaw boards, and sectioned into 20.0 cm long pieces. The wood pieces were submerged in water for complete saturation for 60 days. After this period, a wood particle generator (MA685, Marconi, Piracicaba, Brazil) was used to obtain sheets with an average thickness of 1.5 mm. Subsequently, the sheets were exposed to sunlight for two days and then ground using a hammer mill (A4, Tigre, São Paulo, Brazil) with an 8 mm mesh size to reduce the particle size. The material was then sieved (I-3007, Contenco, São José da Lapa, Brazil) to provide greater homogenization. For use in panel manufacturing, particles that passed through the 4 mm mesh and were retained by the 2 mm mesh were used. These particles were dried in a forced-air oven (SL-102/480, Solab Piracicaba, Brazil) until reaching a final moisture content of 8%. The eggshell waste was crushed and sieved through a 2 mm diameter mesh, and those retained by the 1 mm mesh were utilized.

### 2.2 Production of panels

**Fig. 1:**
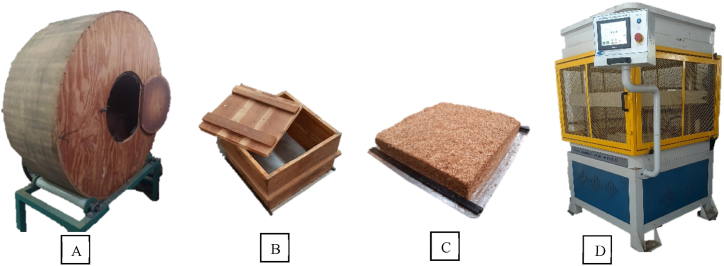
Apparatus for applying glue to wood particles (glue machine) (A); Particle mattress forming wooden box (B); Formed mattress (C); e Electric flat plate press with heating (D).

The processed eucalyptus particles were placed inside a gluing machine (Fig. 1A), and a commercial urea-formaldehyde-based adhesive (Redemite, Cascamite, São Paulo, Brazil) with ammonium sulfate (24%) used as a catalyst (2% based on the solids content of the resin) was applied using a pneumatic gun attached to the machine until the adhesive was fully applied (12 parts based on the dry weight of the particles). Next, the particles bonded with the adhesive were placed in a wooden forming box (42.5 cm × 42.5 cm) (Fig. 1B), and the eggshells were manually added onto the particles after gluing. The proportion was defined in the laboratory together with the team after extensive discussion, considering its density, shape, and granulometry. Eggshells that passed through the 2 mm mesh sieve and were retained on the 1 mm mesh were used.

The mixture was then homogenized and pre-pressed between two aluminum sheets with two 1.2 cm thick spacers, forming a mattress-like structure (Fig. 1C). The formed mattress was placed in a heated hydraulic press (SL-12/150, Solab, Piracicaba, Brazil) and pressed at a temperature of 140 °C under a pressure of 50 tons for 6 min (Fig. 1D). The panels were designed to have a predetermined density of 700 kg/m^3^.

### 2.3 Physical tests

For carrying out the physical tests, for each panel, specimens with dimensions of 50 mm × 50 mm were obtained according to NBR 14810-2 [39].

The physical properties of the panels were characterized through the following tests: bulk density, moisture content, thickness swelling, and water absorption (after 2 and 24 h of immersion). The density profile of the panels was obtained using an X-ray densitometer (DAX 6000, Grecom, Germany). The test specimens were kept in a climatized room at 25 °C ± 2 °C and 65% ± 5% relative humidity for a stabilization period of 30 days to achieve moisture equilibrium.

### 2.4 Mechanical tests

Static bending tests (MOR and MOE), screw withdrawal (SW), and shear strength perpendicular (IB) to the panel were performed. The tests were conducted according to the NBR 14810-2 standard [39]. A universal testing machine with automated data acquisition (EMIC, DL-100KN, Brazil) was used for the tests. The loading rate used was 10 MPa/min (MOR and MOE), and the test specimens were positioned on supports with a clear span of 22.5 cm.

The Janka hardness test was performed based on the D1037-12 standard [40], in a universal testing machine with automated data collection (EMIC, DL-100KN, Brazil). The results obtained were compared with the commercialization standard of the *American National Standards Institute* A208.1 [41], which establishes a minimum value of 22.7 MPa in particleboard panels.

The screw withdrawal resistance test was performed with a tensile force at a speed of 10 mm/min. Perpendicular shear strength was performed at a speed of 2.5 Mpa/min.

### 2.5 Qualitative analysis of the panels

To evaluate the wood-adhesive-eggshell interface, test specimens measuring 1.0 cm × 1.0 cm × 1.2 cm (length, width, and thickness, respectively) were subjected to scanning electron microscopy (JEOL, JSM-IT200, Tokyo, Japan). The specimens were analyzed in the perpendicular region of the panel. Images were obtained in secondary electron (SE) mode at magnifications of 50, 100, and 200 times.

### 2.6 Treatments and statistical analysis of data

The treatments were designed using a completely randomized design using different percentages of eggshells applied to the particles. Three panels were made for each treatment.

Whenever the analysis of variance detected a difference between treatments (p < 0.05) (Table 1), regression was used to analyze the effect of adding eggshell on the evaluated properties.

**Table 1:** Composition of the panels according to the amount of eggshell.

Treatment	Percentage of eggshell on wood particles (dry weight basis)
1	0 - Control
2	1
3	3
4	5
5	10

Statistical analysis was performed using the SISVAR v 5.8 free software [42], with a significance level of 5%.

For the mechanical properties data, correlations were determined, using Pearson’s correlation coefficient, between the percentage of eggshell, MOR, MOE, Janka hardness, screw withdrawal and internal bond.

## 3 Results

### 3.1 Physical properties

Table 2 represents the average values for the density of the panels as well as the compression ratio found and their classification, based on the average density of the panels.

**Table 2:** Mean values for apparent density and compaction ratio of the panels produced as a result of the addition of eggshell.

Treatment	Average density (kg/m^3^)	Compression Ratio	Classification∗
1 (0%)	767.3	1.28	Medium
2 (1%)	769.9	1.27	Medium
3 (3%)	810.3	1.32	High
4 (5%)	818,2	1.31	High
5 (10%)	789.3	1.22	Medium
Average	791,0 (0.084)	1.28 (0.04)	–

∗ Classification according to the A208.1 standard [41]. Value in parentheses is the standard deviation.

**Fig. 2:**
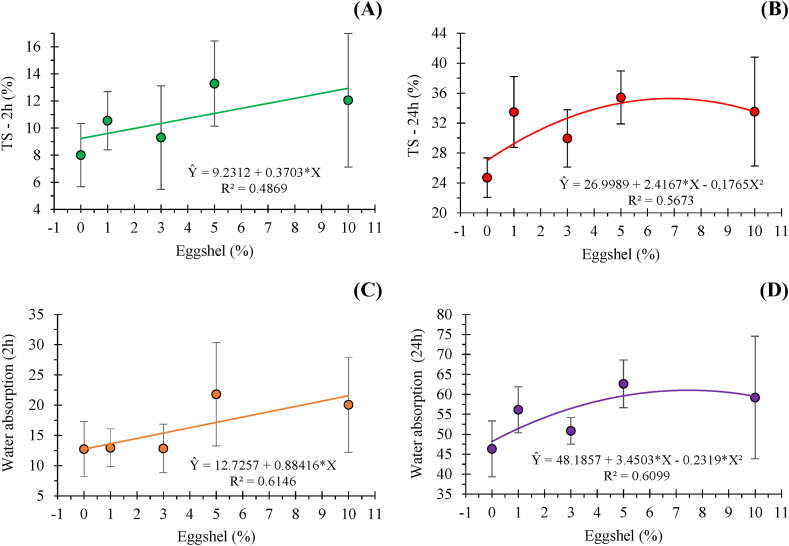
Thickness swelling (TS) after 2 h (A) and 24 h (B) and water absorption after 2 h (C) and 24 h (D) of water immersion of the panels produced with different proportions of eggshell added to eucalypt wood particles.

The behavior of thickness swelling and water absorption in the panels produced are illustrated in Fig. 2.

**Fig. 3:**
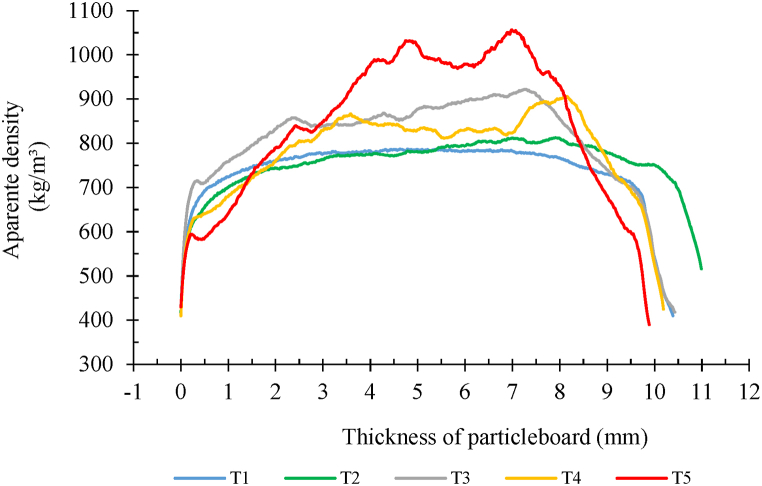
Average representation of the density profile in the treatments studied, based on the addition of eggshell to the eucalypt particles. T1: 0 parts of eggshell (control); T2: 1 part eggshell; T3: 3 parts eggshell; T4: 5 parts eggshell; T5: 10 parts of eggshell on the dry weight of the particles.

Fig. 3 shows the behavior of the density profile performed for the studied panels.

### 3.2 Mechanical properties

The average results obtained in the mechanical tests are shown in Table 3.

**Table 3:** Average results of the mechanical tests of the panels studied.

Treatments	MOR∗ (MPa)	MOE (MPa)	Janka hardness (MPa)	Screw withdrawal (N)	Internal bond (MPa)
1 (0%)	12.84	1578.48	60.99	1046.04	0.64
2 (1%)	7.80	1267.24	52.57	840.53	0.49
3 (3%)	9.75	1582.09	63.35	944.87	0.40
4 (5%)	7.02	1398.77	63.36	813.36	0.40
5 (10%)	6.06	1464.45	66.38	714.51	0.33
Average	8.70 (3.04)	1458.21 (267.93)	63.33 (10.21)	868.31 (216.57)	0.45 (0.17)

∗ MOR: rupture modulus; MOE: elasticity modulus. Value in parentheses is the standard deviation.

With the results of the simple linear correlations between the mechanical characteristics of the particleboards and the apparent density (Table 4), it is possible to verify that there are significant correlations between the modulus of rupture (MOR), Janka hardness, screw withdrawal (SW) and internal bond (IB) with the percentage of eggshell tested.

**Table 4:** Pearson’s simple correlation matrix for mechanical tests.

Evaluated parameter	Eggshell	Apparent density	MOR*	MOE	Hardness	SW	IB
Apparent density	0.109 ^ns^	1					
MOR	−0.381*	−0.050 ^ns^	1				
MOE	0.084 ^ns^	−0.013 ^ns^	0.761*	1			
Janka hardness	0.308*	0.215 ^ns^	0.285*	0.394*	1		
SP	−0.340*	0.056 ^ns^	0.352*	0.193 ^ns^	−0.025 ^ns^	1	
IB	−0.539*	−0.009 ^ns^	−0.184 ^ns^	−0.184 ^ns^	−0.105 ^ns^	0.012 ^ns^	1

* MOR: rupture modulus; MOE: elasticity modulus; SW: screw withdrawal resistance; IB: internal bond; * significant (Pearson, p < 0.05); ^ns^: not significant (Pearson, p > 0,05).

Additionally, there is a significant correlation between the relationships of MOE (elasticity modulus), Janka hardness, and screw withdrawal concerning MOR (rupture modulus). There is also a significant correlation between MOE and Janka hardness.

**Fig. 4:**
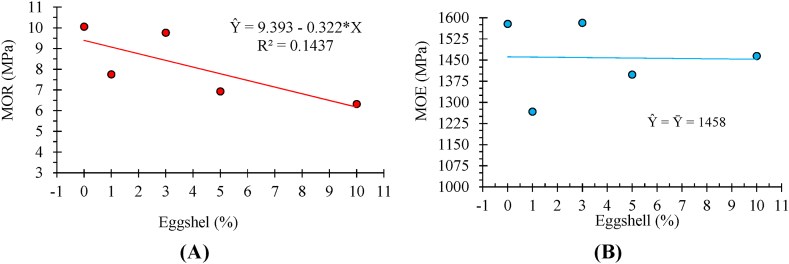
Representation of linearity for static bending - rupture modulus (A) and elasticity modulus (B) as a function of the percentage of eggshell applied to the particleboards.

The results presented in the model for estimating MOR (rupture modulus) about the percentages of eggshell used in panel fabrication (Fig. 4A) show that the highest means are observed in panels made with 100% wood.

**Fig. 5:**
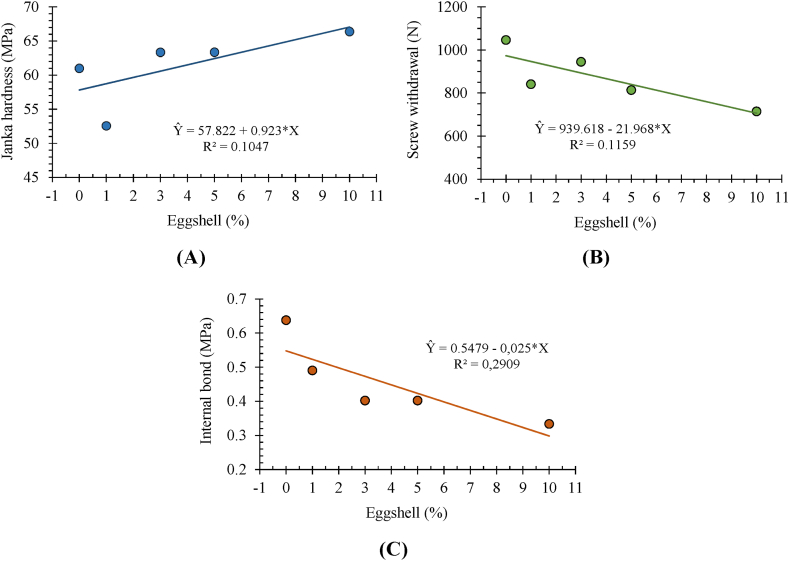
Linear representation between Janka hardness (A), screw withdrawal resistance (B) and internal bond (C) in relation to eggshell proportions.

The MOE showed no significant relationship with the addition of eggshells in the panels (Fig. 4B). Results for Janka hardness, screw withdrawal and internal bond are shown in Fig. 5A–C.

The relationship between internal bond and the proportion of eggshell (Fig. 5C) was linear, in which the addition of shell promoted a reduction in perpendicular tensile strength.

## 4 Discussion

### 4.1 Physical properties

The ANOVA of the regression for humidity and apparent density of the panels showed no statistical difference (F ≥ 0.05), however, the addition of eggshell (T2, T3, T4 and T5) presented numerically higher values, without proportionality. The average moisture content observed in the panels was 10.96% among the five treatments, within the ideal range indicated by the NBR 14810-2 standard [39].

The standard recommends a minimum moisture content of 5% and a maximum of 13% for thicknesses between 10 and 13 mm. With the addition of eggshells, there was a reduction of up to 36% in the moisture content in the panels depending on the percentage of shell added, which is probably due to the porous nature of the eggshell, which is not capable of retaining water [43,44].

The panel compaction ratio showed little variation (1.22–1.32; Table 2), remaining close to the value considered ideal (≈1.3) [43].

Higher thickness swellings were observed in panels that had more eggshell in their composition (T4 and T5) compared to the control, showing a significant and increasing behavior. After 24 h of immersion, all panels with eggshells (T2, T3, T4, and T5) exhibited higher swelling compared to the control, with maximum variations of approximately 5%. The interaction between the adhesive and the materials, together with their geometries, may have directly influenced the results.

Furthermore, the eggshell may have contributed negatively to the adhesion process to the wood particles, as unlike wood, it does not have active sites for bonding with the adhesive and the particles, which facilitates the absorption of water by the particles and adhesive and releasing the tensions formed inside the panel due to pressing.

In general, the addition of eggshell favored water absorption and, consequently, panel swelling, as a higher amount of residue resulted in increased water absorption, especially at higher proportions of eggshell, exceeding the maximum limit specified by the NBR 14810-2 standard [39], which is 16% (Fig. 2). The relationship between the higher water absorption rate and thickness swelling is related to the particle compaction ratio during panel production [43,44].

To estimate the regression model, in the 2 h thickness swelling test (TS2), it was possible to observe the significance of the parameters (p < 0.05) (Fig. 2A). For the 24 h thickness swelling test (TS24), the adjusted quadratic model (Fig. 2B) was significant (p < 0.01).

The results presented for the water absorption test (after 2 and 24 h of water immersion) indicate that after the 2-h immersion, there was a gradual increase in water absorption as the percentage of eggshells increased. Panels T4 and T5, which had a higher concentration, absorbed more water than panels with a lower concentration. After 24 h, a similar result to the 2-h immersion was observed, where the addition of eggshell favored water absorption.

For the 2-h water absorption test, treatments 1, 2, and 3 showed values close to 12%, while treatments 4 and 5 had values close to 20%. The NBR 14810-2 [39] does not provide reference values for this test. Therefore, the maximum water absorption value in 2 h was found in treatment 5, with 10% eggshell, which is similar to the behavior observed in the thickness swelling test. As for the 24-h water absorption, the maximum value was observed in treatment 4.

For the linear and quadratic regression models applied to the 2-h and 24-h water absorption tests, both were found to be significant (p < 0.05) (Fig. 2C and D).

In the evaluation of the density profile using X-ray densitometry (Fig. 3), it can be observed that the treatment without the addition of eggshell (T1) showed a certain level of homogeneity compared to the others, and a similar behavior was observed in the treatment 2.

The remaining treatments showed higher densities, which were directly related to the percentage of eggshells present in their composition. In other words, the higher the percentage of eggshell, the higher the panel density. This observation is also supported by the variation observed across the thickness of the panels due to the addition of eggshells. This could also be attributed to the difficulty in achieving homogeneity of the eggshells in the already bonded particles, as the process was done manually.

The behavior of the density profile is variable depending on the density of origin of the raw material, as also observed by other authors, in which panels were produced with different lignocellulosic materials [13,45,46].

### 4.2 Mechanical properties

As the panel density increases, which is the ratio of mass to volume, the ability of the panel to hold or resist screw withdrawal should ideally increase as well [47], However, this relationship was not observed in the current study (Table 3; Fig. 5B). This occurrence may be associated with the difficulty of adhesion as the eggshell fractions increase, leading to the creation of void spaces, even with similar compactness ratios (Table 2).

The relationship between the density and the mechanical properties of the panels was analyzed and tabulated (Table 3; 4). It was found that the density of the panels did not correlate with the mechanical properties, with significant and negative reductions in MOE and MOR, with increases in density. This implies that flexural strength does not depend only on basic properties, such as density [48], but may be associated with the slenderness index [49] of the particles used, and how the eggshell that does not have a pattern in the length of its fragments, but exerts a negative influence on the panels.

In the analysis of MOE, only Janka hardness (r = 0.394) had a significant effect. With higher proportions of eggshell, there was a slight increase in density, with a reduction in the 10% eggshell proportion, but the linearity behavior was not significant (F_regressão_ < 0.05) (Fig. 4B). On average, the eggshell panels did not reach the minimum values stipulated by the ANSI A208-1 [41] standard, which are 12.8 MPa for MOR and 1943.8 MPa for MOE.

For the internal bond, it was possible to verify that the panels met the requirements of the EN 312 standard [50], of 0.24 MPa. The results are close in the literature [51], between 0.15 and 0.46 MPa. The A 208.1 standard [41] establishes internal bond values between 0.15 and 0.40 MPa, values exceeded in all treatments. The internal bond strength is influenced not only by density but also by other factors, such as panel manufacturing conditions [52]. As mentioned earlier, the increase in eggshell proportion negatively affected this property, similar to the effect observed when adding coconut fibers to eucalypt particles in a gradual manner [53]. However, this does not diminish the use of eggshells due to its environmental benefits, particularly in reducing raw material costs and obtaining eco-efficient particleboard panels [[53], [54], [55]].

The commercial standard ANSI A280.1 specifies a minimum Janka hardness value of 22.7 MPa for particleboard. The average values obtained in this study for the five treatments (61.33 MPa) were higher than those reported in the literature [13,56,57], and still met the commercial standard, with a significant downward trend with increasing eggshell content (Fig. 5A).

Janka hardness is strongly influenced by the pressure and temperature during the manufacturing process [58], as well as by the moisture content of the material [59] and the density of the panel, which is associated with the compaction ratio [60,61]. Since all panels in this study were produced under the same temperature and pressure conditions, their densities (Table 2) did not vary and, therefore, exhibited similar hardness values, with a significant increase as the percentage of eggshell content increased.

### 4.3 Qualitative evaluation by image

**Fig. 6:**
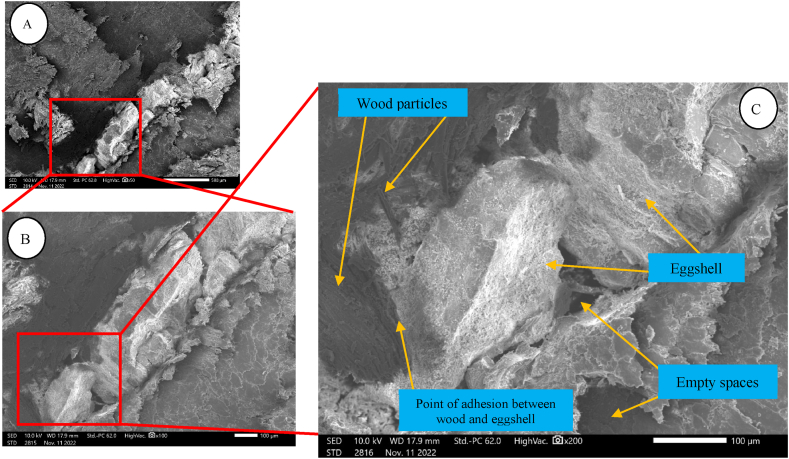
SEM micrograph of eucalyptus wood composite with eggshell particles obtained by scanning electron microscopy. A: 50 times magnification; B: 100x magnification; C: 200x magnification.

In the image generated with scanning electron microscopy (Fig. 6A–C), it is possible to observe the presence of empty spaces in the panels. This observation was possible in all treatments evaluated.

The void content greatly affects the strength of the agglomerate to withstand external loads, therefore, voids are precursors of possible cracks [62,63].

The possible alternative to better fill the spaces in the panels would be to reduce the dimensions of the eggshell particles, which would generate better adhesion to the wood particles, with a greater contact area and reduction of the empty spaces formed [64], since the opt-in mechanisms would be available [53].

In the image, it is possible to observe the eggshell wood particles, where their morphological differences are evident. Finally, an adhesion point was also observed between the wood particle and the eggshell particle, which highlights the existence of interaction between the composite materials.

## 5 Conclusions

The following conclusions have been deduced from the results of the experimental work:

a. The amount of eggshell waste directly influences the density of the panel.
b. The addition of eggshells to the eucalypt particles influenced the increase in water absorption and swelling.
c. The existence of voids in the composite greatly affects the strength of particleboards.
d. The eggshell residue is a feasible raw material alternative for particleboards produced for non-structural purposes, such as furniture.
e. There is a scarcity of published studies that seek to evaluate the combination of eggshell, a widely distributed and abundant agricultural residue, with wood and products derived from this combination. For new alternatives to add value to the product, it is recommended that additional studies be carried out with different adhesives and eggshell particle sizes. In addition to evaluating the resistance of the panels against xylophagous organisms to check whether there is an increase in the resistance of panels produced with different percentages of eggshell. Finally, knowledge about the potential use of eggshells for the production of panels will also bring a great contribution in the environmental sphere due to the correct destination, increase in value and reuse of the waste.

## Data availability

Data will be made available on request.

## CRediT authorship contribution statement

**Vinícius Borges Taquetti:** Writing – original draft, Methodology, Investigation, Data curation. **Vitor Viana Silva:** Writing – original draft, Methodology, Investigation, Data curation. **Izabella Luzia Silva Chaves:** Writing – review & editing. **Rafael Gonçalves Espósito Oliveira:** Writing – original draft, Methodology, Investigation, Data curation. **Fernanda Dalfiôr Maffioletti:** Writing – original draft, Methodology, Investigation, Data curation. **Glaucileide Ferreira:** Writing – original draft, Methodology, Investigation, Data curation. **José Paulo Costa Mendonça:** Writing – original draft, Methodology, Investigation, Data curation. **Emilly Soares Gomes Silva:** Writing – review & editing. **Fabricio Gomes Gonçalves:** Writing – review & editing, Supervision, Formal analysis, Conceptualization.

## Declaration of competing interest

The authors declare that they have no known competing financial interests or personal relationships that could have appeared to influence the work reported in this paper.
